# This is Advertising! Effects of Disclosing Television Brand Placement on Adolescents

**DOI:** 10.1007/s10964-016-0493-3

**Published:** 2016-05-10

**Authors:** Eva A. van Reijmersdal, Sophie C. Boerman, Moniek Buijzen, Esther Rozendaal

**Affiliations:** 1 Amsterdam School of Communication Research (ASCoR), University of Amsterdam, P.O. Box 15791, 1001 NG Amsterdam, The Netherlands; 2 Behavioural Science Institute, Radboud University, Nijmegen, The Netherlands

**Keywords:** Disclosure, Forewarning, Brand placement, Adolescents, Advertising, Persuasion knowledge

## Abstract

As heavy media users, adolescents are frequently exposed to embedded advertising formats such as brand placements. Because this may lead to unwitting persuasion, regulations prescribe disclosure of brand placements. This study aimed to increase our understanding of the effects of disclosing television brand placements and disclosure duration on adolescents’ persuasion knowledge (i.e., recognition of brand placement as being advertising, understanding that brand placement has a persuasive intent and critical attitude toward brand placement) and brand responses (i.e., brand memory and brand attitude). To do so, an earlier study that was conducted among adults was replicated among adolescents aged 13–17 years (*N* = 221, 44 % female). The present study shows that brand placement disclosure had limited effects on adolescents’ persuasion knowledge as it only affected adolescents’ understanding of persuasive intent, did not mitigate persuasion, but did increase brand memory. These findings suggest that brand placement disclosure has fundamentally different effects on adolescents than on adults: the disclosures had less effects on activating persuasion knowledge and mitigating persuasion among adolescents than among adults. Implications for advertising disclosure regulation and consequences for advertisers are discussed.

## Introduction

Adolescents are growing up in an increasingly commercialized media environment (Buijzen et al. 2010). Advertising is omnipresent and is increasingly embedded into editorial content, such as television programs, games, movies, magazine articles, and websites. A popular example of embedded advertising is brand placement, that is, the paid inclusion of branded products or brand identifiers within mass media programming (Karrh 1998). In brand placement, the boundaries between advertising and editorial content disappear, making it unclear whether or not it is actual advertising (Cain 2011; Nebenzahl and Jaffe 1998).

However, according to consumer laws, both minors and adults have the right to know when they are being subjected to advertising (Cain 2011). Brand placement seems to violate these rights because its persuasive intent is masked (Kuhn et al. 2010; Nebenzahl and Jaffe 1998). Therefore, new regulations prescribing disclosure of the commercial nature of embedded forms of advertising including brand placement have been introduced both in Europe and the US to inform audiences (Cain 2011; Nairn and Fine 2008). As a consequence, television programs in the UK and Belgium that include brand placement now show a “PP” logo for “product placement,” and Dutch television programs show texts such as “This program contains product placement.”

Research among adults has shown that disclosures for brand placement can activate persuasion knowledge and mitigate persuasion (e.g., Boerman et al. 2012; Campbell et al. 2013; Tessitore and Geuens 2013; Wei et al. 2008). Persuasion knowledge comprises of consumers’ knowledge and attitudes about persuasion attempts. It consists of several elements, including the recognition of advertising, understanding of persuasive intent and critical attitudes toward advertising (Friestad and Wright 1994; Rozendaal et al. 2011). To date, no research on the effects of disclosure on adolescents has been conducted; however, there are strong reasons to assume that disclosure effects on adolescents are differ significantly from the effects on adults. These reasons relate to certain developmental characteristics of adolescents, including their levels of persuasion knowledge, their immature executive functions and self-regulatory skills, and the importance of identity development and related susceptibility to product and brand symbolism during adolescence. First, although persuasion knowledge continues to develop in adolescence, teenagers still have less mature persuasion knowledge than adults, in particular, when embedded forms of advertising such as brand placements are concerned (Boush et al. 1994; Verhellen et al. 2014). If persuasion knowledge regarding brand placement is not fully developed, disclosures may not be able to activate this knowledge (An and Stern 2011).

Second, because adolescents’ executive functions (e.g., working memory, inhibitory control, attentional flexibility) are still emerging, they experience more difficulties with monitoring and controlling their thoughts, feelings, and actions than adults (Best and Miller 2010; Blakemore and Choudhury 2006; Crone 2009; Moilanen 2007). Therefore, adolescents’ ability to active their (limited) persuasion knowledge and elaborate critically on the commercial intent of a brand placement (i.e., stop and think response; Rozendaal et al. 2011) may be lower.

Third, adolescents are in the midst of identity development in which self-presentation through brands and products is very important as well as conformity to peer group or subculture’s popular brands (Arnett 1995, 2014; Albert et al. 2013; John 1999; Nelson and McLeod 2005; Ritson and Elliott 1999; Rozendaal et al. 2013). Moreover, adolescents tend to be more self-conscious than adults because of the neurobiological changes that occur during this critical developmental period. Thus, adolescents may be especially attracted to branded products that, in their view, provide immediate gratification and/or social status (Pechmann et al. 2005; Steinberg 2005). Therefore, adolescents’ motivation to activate their (limited) persuasion knowledge and elaborate critically on the commercial intent of brand placement may be low. Instead of attending to the meaning of the disclosure, they may rather focus on the social meaning of the brand.

The aim of this study is to test whether sponsorship disclosure can activate adolescents’ persuasion knowledge (i.e., recognition of brand placement as being advertising, understanding that brand placement has a persuasive intent and critical attitude toward brand placement) and whether it affects persuasion (i.e., brand memory and brand attitude). To fulfill this aim, this study replicates one of the first studies on sponsorship disclosure effects (Boerman et al. 2012). We decided to replicate this particular study because it is one of the very few studies that focused on both persuasion knowledge and persuasion effects of disclosures. In addition, the stimulus materials used in Boerman et al. (2012) (i.e., an MTV program focusing on sneakers) are suitable and relevant for teenagers as well.

Replicating this study offers a unique opportunity to compare the effects of sponsorship disclosure on adolescents and adults. Similar to the study of Boerman et al. (2012), this study examines the effects of sponsorship disclosure for brand placement in a television program and studies the role of sponsorship disclosure duration (display of the disclosure for a duration of 3 vs. 6 s). The choice for these durations was based on current regulations that obligate broadcasters to display a disclosure for at least 3 s (Ofcom 2011). To investigate whether this was long enough to inform viewers about brand placement and its persuasive intent, we examined both 3 s and the effects of doubling this time (6 s). Boerman et al. (2012) showed that a 6-s disclosure resulted in more recognition of the brand placement as being advertising (conceptual persuasion knowledge), which in turn resulted in more critical beliefs about the placement (attitudinal persuasion knowledge). Also, a 6-s disclosure led to less positive brand attitudes via higher rates of attitudinal persuasion knowledge. In addition, they showed that sponsorship disclosure increased brand memory regardless of the disclosure duration.

### Effects of Sponsorship Disclosure on Persuasion Knowledge

The persuasion knowledge model postulates that only when people recognize a persuasion attempt can their persuasion knowledge be activated (Friestad and Wright 1994). Consumers can use this knowledge to cope with the persuasion attempt. Coping with persuasion means that people use their persuasion knowledge to decide whether they want to be persuaded or whether they want to resist the persuasion. Because the persuasion attempt is masked in brand placement, a disclosure is expected to help consumers realize that brand placement is actually trying to persuade and not solely to entertain or inform, thus activating consumers’ persuasion knowledge (Cain 2011; Van Reijmersdal et al. 2013).

Specifically, a sponsorship disclosure can increase people’s ability to resist the persuasion attempt by informing them about the nature of the message, which enables them to arm themselves by activating their persuasion knowledge in order to prepare certain defense strategies (e.g., counterarguments). Additionally, a disclosure can increase their motivation to resist by inducing feelings of psychological reactance (Brehm and Brehm 1981). Sponsorship disclosure can cause people to feel restricted in their freedom to feel and think what they want. As a consequence, they are motivated to actively restore this freedom (Fransen and Fennis 2014).

According to Rozendaal et al. (2011), there is a distinction between conceptual and attitudinal persuasion knowledge. Conceptual persuasion knowledge is a cognitive construct and is defined as people’s knowledge about persuasion. Conceptual persuasion knowledge includes several components, such as the recognition of a message as being advertising and the understanding of advertising’s persuasive intent and tactics. Legislators aim to inform consumers about the commercial nature of advertising so that consumers can distinguish advertising from editorial content and recognize the persuasive intent (Cain 2011), thus activating conceptual persuasion knowledge. Boerman et al. (2012) focused only on the recognition of advertising, but following the aims of disclosures, the present study also includes the understanding of persuasive intent.

The second construct of persuasion knowledge is attitudinal and is defined as people’s critical attitudes toward persuasion. It involves critical beliefs about honesty, credibility, and trustworthiness of the persuasion attempt (Rozendaal et al. 2011). Sponsorship disclosure may affect attitudinal persuasion knowledge as a result of the activation of conceptual persuasion knowledge. When consumers realize that brand placement is advertising and has a persuasive intent, this may trigger critical beliefs and activate attitudinal persuasion knowledge (Boerman et al. 2012). When they realize that a persuasion attempt is made, consumers become critical and show reactance to preserve their freedom (Brehm and Brehm 1981).

Boerman et al. (2012) indeed found that disclosures activated conceptual persuasion knowledge (i.e., better recognition of brand placement as being advertising), which in turn resulted in higher scores for attitudinal persuasion knowledge (i.e., more critical beliefs about the brand placement). Similarly, other studies among adults have shown that sponsorship disclosure activated persuasion knowledge (e.g., Boerman et al. 2012; Boerman et al. 2014; Wood et al. 2008).

The question remains whether these effects of sponsorship disclosure on conceptual and attitudinal persuasion knowledge also hold for adolescents. On the one hand, we expect that adolescents will benefit from sponsorship disclosure. That is, theories on children’s and adolescents’ advertising processing (Buijzen et al. 2010; Rozendaal et al. 2011), in which a developmental perspective on adult persuasion models is adopted, suggest that due to the embedded nature of advertising formats such as brand placements, children and adolescents primarily process embedded advertising formats under conditions of low cognitive elaboration. As a consequence, when confronted with a brand placement, they are unlikely to autonomously activate their persuasion knowledge and use it to critically evaluate the brand placement. In other words, they are unlikely to “stop and think” about the commercial purpose of a brand placement (Rozendaal et al. 2011). It is expected that a sponsorship disclosure can trigger such a “stop and think” response, thereby increasing adolescents’ ability to activate their persuasion knowledge and to critically evaluate the brand placement.

On the other hand, it is debated whether adolescents’ persuasion knowledge has already fully matured (Boush et al. 1994; Moschis and Churchill 1978; Nairn and Fine 2008). For example, Rozendaal et al. (2010) showed that, in early adolescence, children had the same level of understanding advertising’s selling intent but that their grip of persuasive intent was not at an adult level. In addition, Boush et al. (1994) showed that adolescents’ understanding of advertiser tactics was significantly lower than that of adults. This implies that some elements of persuasion knowledge are not fully matured, indicating limited levels of persuasion knowledge among adolescents. Moreover, adolescents’ recognition of brand placement as being commercial has been found to be even more limited due to its hidden nature (Verhellen et al. 2014). Having persuasion knowledge is a necessary precondition for a sponsorship disclosure to be effective, because it is only when adolescents understand the commercial intent of brand placements that a disclosure will be able to activate this knowledge. Adolescents’ limited persuasion knowledge, in particular with regard to embedded advertising formats such as brand placement, may therefore limit the effectiveness of sponsorship disclosures.

The effectiveness of a sponsorship disclosure may be further limited due to the immature levels of executive functions that characterize adolescence, in particular, inhibitory control and cognitive flexibility abilities (Best and Miller 2010; Blakemore and Choudhury 2006; Crone 2009). Inhibitory control involves the ability to control one’s thoughts, emotions, and behavior to override an impulsive response and instead do what is more appropriate (Diamond 2012). Cognitive flexibility is the mental ability to shift one’s attention in response to changing goals or environmental stimuli (Diamond 2012). Due to immature levels of inhibitory control and cognitive flexibility, adolescents may be more likely to immediately respond to the salient and emotionally pleasing features of a brand placement and less likely to activate a critical manner of processing (Rozendaal et al. 2011), even when a sponsorship disclosure is present.

### Effects of Sponsorship Disclosure on Brand Responses

Although legislators and regulators primarily aim to help consumers activate their persuasion knowledge with disclosures (i.e., increase the recognition of advertising and understanding of the persuasive intent of brand placement), the literature has shown that there are effects on persuasion as well. The majority of studies showed that exposure to a sponsorship disclosure increased memory of brand placements because the disclosure serves as a prime to focus on the brand (Boerman et al. 2012, 2014; Van Reijmersdal et al. 2013; Wood et al. 2008). Based on insights on adolescents’ developmental characteristics, there are no reasons to assume that the brand memory of adolescents is affected differently by the disclosure compared to the brand memory of adults. Therefore, as for adults, disclosures are expected to have positive effects on adolescents’ brand memory. For them, the disclosure may also serve as a prime to focus on the brand, increasing the chance of brand memory.

Previous studies have also focused on how sponsorship disclosure affects brand attitudes, and mixed results have been found. Some studies failed to demonstrate (direct) sponsorship disclosure effects on attitudes toward brands placed in television programs and movies (Dekker and Van Reijmersdal 2013; Matthes and Naderer 2016; Van Reijmersdal et al. 2015). However, Boerman et al. (2012) demonstrated that a disclosure negatively affected attitudes toward a brand placed in a television program but only indirectly via attitudinal persuasion knowledge. That may explain why other studies did not find direct effects of sponsorship disclosure. Due to the disclosure, consumers activated their critical attitudes toward the brand placement, which they consequently used to resist the persuasion and to establish negative brand attitudes. Similarly, Campbell et al. (2013) found negative effects of sponsorship disclosure on brand attitudes.

It is unclear whether sponsorship disclosure can influence brand attitudes among adolescents. As with adults, a disclosure might trigger critical attitudes toward the brand placement and lead to more negative brand attitudes. However, there are reasons to expect that, for adolescents, this may not be the case. As was argued earlier, the effect of a sponsorship disclosure may be limited due to the relatively low levels of inhibitory control and cognitive flexibility that characterize adolescence (Best and Miller 2010; Blakemore and Choudhury 2006; Crone 2009). Due to their immature inhibition and cognitive flexibility, adolescents may be more likely to be swayed by entertaining and emotionally appealing features of a brand placement and less likely to activate a critical manner of processing (Rozendaal et al. 2011), even when a sponsorship disclosure is present. This would indicate that disclosures of sponsorship have no effect on adolescents’ brand attitudes. A disclosure could even have a positive effect on brand attitude because increasing adolescents’ awareness of the brand could strengthen the positive affect transfer of context to brand.

Furthermore, self-presentation and conformity to a subculture or to peers are crucial for adolescents to create their own identity and their need to fit in (Brown 1990; Grotevant 1987). For example, as compared to adults, adolescents are more sensitive to social status based on brand use, image, and physical appearance (Buijzen et al. 2010; Lui et al. 2014; Pechmann et al. 2005; Ritson and Elliott 1999). Therefore, the portrayal of brands in entertaining and involving contexts may have a strong influence on adolescents. Nelson and McLeod (2005) indeed found that adolescents score high on awareness of brand placements and that brand consciousness was positively related to attention and favorable attitudes to brand placement.

These effects may overrule any possible negative effects of sponsorship disclosure on brand attitudes. Even if disclosures activate persuasion knowledge among adolescents, their susceptibility to consumer symbolism may supersede resistance to persuasion and the formation of negative brand attitudes.

### Effects of Sponsorship Disclosure Duration

Boerman et al. (2012) showed that the duration of sponsorship disclosure determines its effectiveness. They showed that a 6-s disclosure resulted in more negative brand attitudes through attitudinal persuasion knowledge than a 3-s disclosure. This effect can be explained by limited capacity models of information processing (Buijzen et al. 2010; Lang 2000). These models postulate that consumers’ cognitive resources to process information are limited. Therefore, consumers are selective in allocating their cognitive resources. While watching a television program, consumers are exposed to many elements of information at the same time; in the case of the current study, these elements are (a) the story line, (b) the brand placement, and (c) the disclosure (Boerman et al. 2015). Because of consumers’ limited cognitive capacities, it is impossible to attend to all these elements at the same time. Extending the duration of the disclosure from 3 to 6 s may provide consumers with more opportunity to allocate their cognitive resources to the disclosure and increases the chance that it has an effect.

For adolescents, similar effects may be expected. Because of their limited cognitive processing skills, adolescents are expected to be in even greater need of enhanced opportunities to process sponsorship disclosures. That is, due to their immature executive function skills, in particular, their cognitive flexibility and working memory, adolescents have more difficulties in focusing their attention and in processing distinct pieces of information at the same time (Best and Miller 2010; Blakemore and Choudhury 2006; Crone 2009). The richness of information presented in a television program that also includes brand placement and disclosures may make it difficult for adolescents to process the disclosure.

However, a longer duration of the disclosure may provide adolescents with the extra opportunity to process the information in the disclosure that is needed for it to activate adolescents’ limited persuasion knowledge and to exert an effect on persuasion. Therefore, it is expected that disclosure duration has a positive effect on both conceptual and attitudinal persuasion knowledge as well as on brand memory and brand attitudes.

## The Current Study

Adolescents are increasingly exposed to brands embedded within entertainment media. Because this may lead to unwitting persuasion, regulations prescribe disclosure of brand placements (i.e., sponsorship disclosure). Yet, it remains unknown whether or not sponsorship disclosure is actually effective in increasing adolescents’ awareness of this advertising practice and whether it can change the way they are persuaded by it. Therefore, the aim of this study is to test whether sponsorship disclosure can activate adolescents’ persuasion knowledge (i.e., recognition of brand placement as being advertising, understanding that brand placement has a persuasive intent and critical attitude toward brand placement) and whether it affects persuasion (i.e. brand memory and brand attitude).

To fulfill this aim, this study replicates one of the first studies on sponsorship disclosure effects (Boerman et al. 2012). Following Boerman et al., this study focuses on the effects of sponsorship disclosure for brand placement in a television program and will examine the effects of the sponsorship disclosure duration (display of the disclosure for a duration of 3 vs. 6 s). By replicating Boerman et al., this study offers insights into the differential effects of sponsorship disclosure on adolescents and adults. Research among adults has shown that disclosures for brand placement can activate persuasion knowledge (i.e., people’s theories about persuasion and their beliefs about marketers’ motives, and strategies) and mitigate persuasion. However, based on insights on adolescent development, there are strong reasons to assume that disclosure effects on adolescents are fundamentally different than with adults. These reasons have to do with adolescents’ levels of persuasion knowledge, their immature executive function skills (i.e., working memory, inhibition, and cognitive flexibility), and the importance of identity development and related susceptibility to product and brand symbolism. Although we expect certain differences between adults and adolescents to exist, we decided to formulate the same hypotheses as Boerman et al. because this will enable us to make a better comparison between the current study and the one of Boerman et al.

Boerman et al. (2012) hypothesized that because the persuasion attempt is masked in brand placement, a disclosure can help consumers realize that brand placement is actually trying to persuade and not solely to entertain or inform, thus activating consumers’ conceptual (i.e., better recognition of brand placement as being advertising) and attitudinal persuasion knowledge (i.e., more critical beliefs about the brand placement). Although the effect of sponsorship disclosure on persuasion knowledge may be different for adolescents because they have lower levels of persuasion knowledge and less developed executive functions skills than adults, we followed Boerman et al. (2012).

Therefore we expect that sponsorship disclosure (compared to no disclosure) leads to the activation of adolescents’ conceptual persuasion knowledge, that is, (a) recognition of the brand placement as being advertising and (b) understanding of the persuasive intent of the brand placement (Hypothesis 1). Furthermore, we hypothesize that sponsorship disclosure (compared to no disclosure) leads to the activation of adolescents’ attitudinal persuasion knowledge (i.e., critical attitude toward the brand placement; Hypothesis 2). In addition, we expect that adolescents’ conceptual persuasion knowledge mediates the effect of sponsorship disclosure on attitudinal persuasion knowledge such that a disclosure activates conceptual persuasion knowledge, which leads to the activation of attitudinal persuasion knowledge (Hypothesis 3).

Boerman et al. (2012) hypothesized that a sponsorship disclosure can also lead to increased memory of brand placements because the disclosure serves as a prime to focus on the brand. There are no developmental reasons to expect this effect will be different for adolescents. Therefore, we expect that sponsorship disclosure (compared to no disclosure) leads to higher brand memory among adolescents (Hypothesis 4). In addition, Boerman et al. (2012) hypothesized that disclosures can have a negative effect on brand attitudes, and this effect is a result of the activation of persuasion knowledge, which is consequently used to resist the persuasion and to establish negative brand attitudes. Due to adolescents’ immature executive functions (primarily inhibitory control and cognitive flexibility) and the importance of brand symbolism for their identity development, this effect might be absent or even positive for adolescents. However, for the sake of comparison, we follow Boerman et al. and expect that sponsorship disclosure (compared to no disclosure) has a negative effect on adolescents’ attitude toward brands placed in a television program (Hypothesis 5). Furthermore, we hypothesize that the effect of sponsorship disclosure on adolescents’ brand attitude is mediated by (a) conceptual persuasion knowledge and (b) attitudinal persuasion knowledge, such that a disclosure activates conceptual persuasion knowledge and attitudinal persuasion knowledge and both in turn lead to more negative brand attitudes (Hypothesis 6).

Finally, Boerman et al. (2012) showed that disclosure duration has a positive effect on persuasion knowledge as well as on brand memory and brand attitudes, meaning that a 6-s disclosure is more effective than a 3-s disclosure. Based on insights on the executive function skills of adolescents, we expect the same effect to occur among adolescents. Thus, a longer duration of sponsorship disclosure results in higher (a) activation of conceptual persuasion knowledge, (b) attitudinal persuasion knowledge, (c) brand memory and (d) more negative brand attitudes (Hypothesis 7).

## Methods

### Sample and Procedure

An experiment was conducted with three conditions: a control group (*n* = 32) and two disclosure conditions (disclosure displayed for 3 s, *n* = 95, or 6 s *n* = 94). The current study was conducted in the fall of 2012 in the same country as the original study by Boerman et al. (2012), for which data were collected in the summer of 2011. A total of 221 adolescents (56 % male) between the age of 13 and 17 years old (*M* = 15.21, *SD* = 0.94) participated in the study. Most respondents were 15 or 16 years old (78 %), and all adolescents followed higher general secondary education or pre-university education. Before the research started, it was granted IRB (institutional review board) approval. Active informed consent was obtained from the adolescents and the heads of the schools, and passive informed consent was obtained from the parents. Respondents received no incentive, and we randomly assigned them to one of the experimental conditions. The experiment was an exact replication of the study conducted by Boerman et al. (2012) using the same stimulus materials and the same measures, only now the study was administered among adolescents and included a measure of understanding persuasive intent (Lapierre 2015).

During the experiment, which lasted about 30 min, participants sat in a classroom behind an individual computer with headphones on. First, they watched the episode containing brand placement. At the end of the show, participants filled out a questionnaire including questions about the program (program familiarity, program viewing frequency, and program involvement), followed first by questions about the brand (recall, attitude, familiarity, and use), and then conceptual and attitudinal persuasion knowledge. Next, they answered questions about their memory of the sponsorship disclosure and their demographics.

### Stimuli

We asked participants to watch an episode from the weekly television program *MTV Was Here*, which includes three items on lifestyle, gadgets, or music. For the experiment, a professional created a new episode of 14 min by merging three items from three episodes. These episodes were originally aired in the spring of 2011. The structure of the program was similar to the original episodes.

In the item in the middle (4 min and 20 s), the presenter of the show introduces a new shoe brand, Alive Shoes, visits the store, and talks to the creator of the shoes. The brand was mentioned seven times, and the brand and the sneakers were visible either in the background or more prominently for 1 min and a half in total.

The disclosure that was displayed for either 3 or 6 s in the experimental conditions stated the following: “This program contains advertising by Alive Shoes.” The duration was based on regulations that obligate broadcasters to display a disclosure for at least 3 s (Ofcom 2011), and the wording was following regulation proposals in the United States (Cain 2011; Ong 2011).^1^ The disclosure appeared in the upper right corner of the screen and was clearly readable since its size was comparable to the size of common subtitles (covering approximately 2.5 % of the screen).

### Measures

#### Conceptual Persuasion Knowledge

We measured the two aspects of conceptual persuasion knowledge with four items. We measured *Recognition of Advertising* by asking participants to what extent (1 = *strongly disagree*, 7 = *strongly agree*) they believed the item about Alive Shoes was advertising (*M* = 5.43, *SD* = 1.48; Boerman et al. 2012; Ham et al. 2015)

To measure *Understanding of Persuasive Intent,* we asked participants to indicate the extent to which they thought the item about Alive Shoes was made to “make you like the brand”, “sell Alive Shoes”, and “influence you” (1 = *strongly disagree*, 7 = *strongly agree;* Boerman et al. 2012). Factor analysis revealed the items’ load on one factor (Eigenvalue = 1.88; explained variance = 62.75 %; Cronbach’s alpha = .70). The mean score of the three items is used as a measurement of the understanding of persuasive intent (*M* = 5.31, *SD* = 1.19).

#### Attitudinal Persuasion Knowledge

To measure their attitudinal persuasion knowledge, we asked participants to what extent they agreed (1 = *strongly disagree*, 7 = *strongly agree*) with the statement, ‘I think the item about Alive Shoes in *MTV Was Here* is… “honest” (reversed), “trustworthy” (reversed), “convincing” (reversed), and “not credible.”’ Attributes were based on a scale measuring source trustworthiness (Boerman et al. 2012; Ohanian 1990). Although factor analysis revealed that the four items’ load on one factor (Eigenvalue = 1.94; explained variance = 48.53 %), leaving out the item “not credible” (Cronbach’s alpha increased from .60 to .69), improved the reliability of the scale. The reverse coding may have confused the adolescents. High scores of attitudinal persuasion knowledge correspond to more critical feelings, whereas low scores correspond to less critical feelings (*M* = 3.73, *SD* = 1.18).

#### Brand Memory

Participants indicated whether they recalled seeing any brands in the episode of *MTV Was Here* and, if so, which brands (1 = *mentioned Alive Shoes,* 0 = *did not mention* *Alive Shoes;* 32 % brand recall).

#### Brand Attitude

To tap into participants’ attitudes toward the brand, we used six 7-point semantic differential scales: bad/good, unpleasant/pleasant, unfavorable/favorable, negative/positive, dislike/like, and poor quality/high quality (Bruner et al. 2001). Factor analysis revealed that the items load on one factor (Eigenvalue = 4.01; explained variance = 66.90 %; Cronbach’s alpha = .90). We used the mean score of the six items as a measurement of brand attitude (*M* = 4.15, *SD* = 1.21).

#### Control Variables

We measured several control variables (identical to the original study; Boerman et al. 2012) to make sure that the effects of the disclosure and its duration were not caused by other differences between the experimental groups.

##### Program Familiarity

Participants were asked whether they were familiar with the television program *MTV Was Here* (0 = *no*, 1 = *yes*; 53 % knew the program).

##### Program Watching Frequency

Participants indicated how often they watched *MTV Was Here* (1 = never, 2 = less than once a month, 3 = 2–3 times a month, 4 = weekly, 5 = daily). Of the adolescents who were familiar with the program, 27 % never watched it, 39 % watched it (less than) once a month, 23 % watched it 2–3 times a month, and 11 % watched it weekly.

##### Program Involvement

Ten 7-point semantic differential scales from Zaichkowsky’s (1994) personal involvement inventory were used to measure involvement with the *MTV Was Here* program (Eigenvalue = 4.10; explained variance = 51.23 %; Cronbach’s alpha = .86; *M* = 3.98, *SD* = 0.88).

##### Brand Familiarity

Participants were asked whether they were familiar with the brand *Alive Shoes* before this study (97 % was not).

##### Brand Ownership

Participants were asked whether they owned Alive Shoes (none did).

##### Product Interest

We measured product interest by asking participants to indicate to what extent they agreed with the following items (1 = *strongly disagree*, 7 = *strongly agree)*: “I like buying shoes,” “I like to watch programs about shoes on television,” and “I am interested in shoes” (Van Reijmersdal et al. 2007; Eigenvalue = 2.24; explained variance = 74.66 %; Cronbach’s alpha = .83; *M* = 4.15, *SD* = 1.63).

##### Disclosure Recall and Recognition

To ensure that participants noticed the sponsorship disclosure, we asked those who were exposed to a disclosure: “Did you see a disclosure in the program and if so which one?” with the following answering options: the correct disclosure (28 %), three disclosures that were not in the program (together 12 %) and “I did not see any disclosure” (60 %).

### Statistical Analyses

In their study, Boerman et al. (2012) decided to only focus on those participants who remembered seeing the disclosure. To get a complete picture of sponsorship disclosure effects on adolescents, this paper tests all hypotheses and research questions for the total sample and also for a subsample including only those adolescents who remembered seeing the disclosure (subsample *N* = 84: control group *n* = 32, 3-s disclosure *n* = 21, 6-s disclosure *n* = 31). To increase power and reduce the number of individual statistical tests, we used MANCOVA instead of ANCOVA when the dependent variables were correlated, and we ran mediation tests including both mediators instead of using a separate mediation test for each mediator.

To examine which variables should be used as covariates, we used analyses of variance for the continuous variables and analyses of dependence (χ^2^) for the dichotomous variables first for the total sample and next for the subsample.

To test the effects of disclosures on persuasion knowledge (H1 and H2), we performed MANCOVA with the three measures of persuasion knowledge (recognition of advertising, understanding of persuasive intent, and attitudinal persuasion knowledge) as dependent variables and no disclosure versus disclosure (combining the 3- and 6-s disclosures) with a between-subjects factor and age as a covariate.

To test H3, we used the PROCESS macro (model 4, Hayes 2013) with disclosure versus no disclosure as the independent variable, the two measures of conceptual persuasion knowledge as mediators in parallel, and attitudinal persuasion knowledge as the dependent variable. For H4 on the effect of disclosures on brand memory, we conducted a test of dependence (χ^2^) and for H5, we performed ANCOVA with disclosure versus no disclosure as the between-subjects factor, brand attitude as the dependent variable, and age as the covariate. For H6, we ran mediation analyses with the PROCESS macro (model 4, Hayes 2013) and the measures of persuasion knowledge as mediators in parallel, brand attitude as the dependent variable, and age as a covariate.

To test the effects of disclosure duration (H7), we performed the same analyses as above but with the three experimental conditions (no disclosure, 3-s disclosure, 6-s disclosure) as independent variables. For the mediation analyses, we used PROCESS (model 4, Hayes 2013) with the disclosure duration as a dummy variable (3- and 6-s disclosure). We ran all analyses for both the total sample and for the subsample. In the analyses of the subsample, we included only the participants who recognized the disclosure versus participants in the control condition. We tested all effects as one-tailed.^2^

## Results

### Randomization

The three experimental groups (no disclosure, 3-s disclosure, and 6-s disclosure) did not differ with respect to gender, χ^2^(2) = 1.51, *p* = .471; age, *F*(2, 218) = 0.62, *p* = .539; program familiarity, χ^2^(2) = 3.63, *p* = .163; program viewing frequency, χ^2^(6) = 7.64, *p* = .265, program involvement, *F*(2, 218) = 0.13, *p* = .880, brand familiarity, χ^2^(2) = 1.41, *p* = .494; and product interest, *F*(2, 218) = 1.88, *p* = .156. Within the subsample, there were also no significant relations between the experimental groups and the control variables (all *p* > .15). Although the groups did not differ with respect to age, we did include age as a covariate in all analyses to control for any possible effects. Age is an important factor in persuasion knowledge and persuasion (Wright et al. 2005).

### Effects on Persuasion Knowledge

#### Total Sample

First, we tested whether disclosure leads to increased conceptual persuasion knowledge (i.e., recognition of advertising and understanding of persuasive intent) and increased attitudinal persuasion knowledge (H1 and H2). MANCOVA showed no significant effects of a disclosure on the recognition of advertising, *F*(1, 218) = 0.08, *p* = .39, η^2^ = .00), understanding of persuasive intent, *F*(1, 218) = 1.96, *p* = .08, η^2^ = .01, and attitudinal persuasion knowledge, *F*(1, 218) = 0.00, *p* = 0.50, η^2^ = .00 (see also Table 1). With respect to H3 on the mediating role of conceptual persuasion knowledge in the effect of disclosure on attitudinal persuasion knowledge, results of indirect effects analysis showed no indirect effect of the disclosure on attitudinal PK via the recognition of advertising [indirect effect = −0.002, boot SE = 0.02, 90 % BCCI (−.05; .02)] or understanding of persuasive intent [indirect effect = 0.03, boot SE = 0.03, 90 % BCCI (−.001; .12)].Thus, the data of the total sample do not support H1, H2, or H3.

**Table 1:** Direct effects of sponsorship disclosure on persuasion knowledge and brand responses

	No disclosure (*n* = 32)	Disclosure (all participants: *n* = 189)	Disclosure (subsample: n = 52)
Recognition of advertising	5.50 (1.81)	5.42 (1.43)	5.38 (1.37)
Understanding of persuasive intent	5.05 (1.26)	5.35 (1.81)	5.51 (0.96)^a^
Attitudinal persuasion knowledge	3.76 (1.20)	3.73 (1.18)	3.88 (1.22)
Brand memory	9 %	35 %^a^	27 %^a^
Brand attitude	4.13 (1.02)	4.15 (1.24)	4.15 (1.12)

Separate analyses were performed to compare the no disclosure group to the disclosure group including all participants and to compare the no disclosure group to the disclosure group of the subsample. The subsample includes only respondents who recognized the disclosure

Mean scores with standard deviations between parentheses are presented

^a^Means with a superscript differ significantly from the no disclosure condition at *p* < .05

#### Subsample

For the subsample, the MANCOVA showed no significant effects of a disclosure on the recognition of advertising, *F*(1, 81) = 0.12, *p* = .37, η^2^ = .00, and attitudinal persuasion knowledge, *F*(1, 81) = 0.24, *p* = .32, η^2^ = .00 (see means in Table 1). However, there was a significant effect on the understanding of persuasive intent, *F*(1, 81) = 3.59, *p* = .03, η^2^ = .04. This means that compared to no disclosure, showing a disclosure increased adolescents’ understanding of the persuasive intent of the brand placement when they remembered seeing the disclosure. Thus, the data supported the hypothesized positive effect of disclosure on understanding of persuasive intent among the subsample (H1).

With respect to the mediated effect of disclosure on attitudinal persuasion knowledge via conceptual persuasion knowledge (H3), results of indirect effects analysis showed no indirect effect of the disclosure on attitudinal PK via the recognition of advertising [indirect effect = −0.01, boot SE = 0.04, 90 % BCCI (−.14; .05)] or understanding of persuasive intent [indirect effect = −0.00, boot SE = 0.06, 90 % BCCI (−.13; .11)]. This means we did not find any support for the mediation proposed in H3.

### Effects on Brand Memory

#### Total Sample

H4 predicted a positive effect of a disclosure on adolescents’ brand recall. The results (see Table 1 for percentages) showed a significant positive effect of the disclosure on brand memory, χ^2^(1) = 8.60, *p* = .002. This means that participants who were exposed to a sponsorship disclosure were four times more likely to recall the brand than participants who were not exposed to a disclosure.

#### Subsample

The analysis among the subsample showed the same significant positive effect of the disclosure on brand memory, χ^2^(1) = 3.78, *p* = .03 (see Table 1 for percentages). Participants who recognized seeing the sponsorship disclosure were almost three times more likely to recall the brand than participants who were not exposed to a disclosure. This means that the data support the hypothesized positive effect of disclosure on brand memory (H4) in both samples.

### Effects on Brand Attitude

#### Total Sample

H5 predicted that disclosure would have a negative effect on brand attitude. ANCOVA showed no significant effect of the disclosure on brand attitude, *F*(1, 218) = 0.04, *p* = .42, η^2^ = .00 (see Table 1). With respect to H6 regarding the mediation effect of the disclosure on brand attitude via persuasion knowledge, results revealed no indirect effect of the disclosure on brand attitude via the recognition of advertising [indirect effect = −0.02, boot SE = 0.02, 90 % BCCI (−.04; .02)], understanding of persuasive intent [indirect effect = 0.01, boot SE = 0.02, 90 % BCCI (−.01; .07)], or attitudinal persuasion knowledge [indirect effect = 0.00, boot SE = 0.12, 90 % BCCI (−.20; .21)].

#### Subsample

For the subsample, the analysis showed no significant effect of the disclosure on brand attitude: *F*(1, 81) = 0.00, *p* = .49, η^2^ = .00. This means that H5 on the positive effect of disclosure on brand attitude was not supported in both samples.

With respect to H6 on the mediated effect of disclosure on brand attitude via persuasion knowledge, results revealed no indirect effect of the disclosure on brand attitude via the recognition of advertising [indirect effect = −0.01, boot SE = 0.03, 90 % BCCI (−.11; .04)], understanding of persuasive intent [indirect effect = 0.01, boot SE = 0.04, 90 % BCCI (−.12; .06)], or attitudinal persuasion knowledge [indirect effect = −0.06, boot SE = 0.13, 90 % BCCI (−.34; .18)]. This means we found no evidence of the mediation effect proposed in H6 in the subsample.

### Effect of Disclosure Duration

#### Total Sample

H7 predicted that a longer duration of the disclosure would lead to higher conceptual and attitudinal persuasion knowledge and higher levels of brand attitude, but to more negative brand attitudes. MANCOVA revealed no significant effects of duration on the recognition of advertising, *F*(2, 217) = 0.34, *p* = .36, η^2^ = .00; understanding of persuasive intent, *F*(2, 217) = 1.39, *p* = .13, η^2^ = .01; and attitudinal persuasion knowledge, *F*(2, 217) = 0.20, *p* = .41, η^2^ = .00 (see Table 2).

**Table 2:** Direct effects of disclosure duration (total sample, *N* = 221)

	Total sample		
	No disclosure (*n* = 32)	3-s disclosure (*n* = 95)	6-s disclosure (*n* = 94)
Recognition of advertising	5.50 (1.81)	5.51 (1.38)	5.34 (1.48)
Understanding of persuasive intent	5.05 (1.26)	5.28 (1.11)	5.42 (1.25)
Attitudinal persuasion knowledge	3.76 (1.20)	3.79 (1.20)	3.66 (1.16)
Brand memory	9 %^a^	34 %^b^	37 %^b^
Brand attitude	4.13 (1.02)	4.07 (1.30)	4.23 (1.18)

Mean scores with standard deviations between parentheses are presented

^a,b^Means with different superscripts differ significantly from each other at *p* < .05

Regarding brand memory, the analysis showed a significant effect of duration: χ^2^(2) = 8.87, *p* = .01. To see which conditions differed significantly from each other, a logistic regression was run with disclosure duration as a categorical predictor and the no disclosure condition as reference category. The results revealed a significant effect of both the 3-s (*b* = 1.60, *OR* = 4.95, *p* = .01) and the 6-s disclosure (*b* = 1.76, *OR* = 5.82, *p* = .003) compared to no disclosure [−2 Log Likelihood = 265.24, Nagelkerke R^2^ = .07, χ^2^(3) = 10.74, *p* = .01]. Moreover, the same analysis but with the 6-s disclosure as reference category showed that the 3- and 6-s disclosures did not differ significantly (*b* = −0.16, *OR* = 0.85, *p* = .30). This means that the disclosure had a significant positive effect on brand recall, regardless of its duration.

With respect to the direct effect on brand attitude, ANCOVA revealed no significant effect of the disclosure duration: *F*(1, 217) = 0.30, *p* = .37, η^2^ = .00. Altogether, this means that H7 is not supported in the total sample: disclosure duration did not affect persuasion knowledge, brand memory, or brand attitude.

#### Subsample

For the subsample, MANCOVA revealed no significant effects on recognition of advertising, *F*(2, 80) = 1.24, *p* = .15, η^2^ = .03, understanding of persuasive intent, *F*(2, 80) = 1.77, *p* = .09, η^2^ = .04, and attitudinal persuasion knowledge, *F*(2, 80) = 0.15, *p* = .43, η^2^ = .00 (see Table 3).

**Table 3:** Direct effects of disclosure duration for the subsample (only those who recognized the disclosure (*N* = 84)

	No disclosure (*n* = 32)	3-s disclosure (*n* = 21)	6-s disclosure (*n* = 31)
Recognition of advertising	5.50 (1.81)	5.71 (1.15)	5.16 (1.49)
Understanding of persuasive intent	5.05 (1.26)	5.54 (0.90)	5.49 (1.01)
Attitudinal persuasion knowledge	3.76 (1.20)	4.03 (1.29)	3.77 (1.18)
Brand memory	9 %^a^	14 %^a^	36 %^b^
Brand attitude	4.13 (1.02)	4.09 (1.00)	4.19 (1.20)

Mean scores with standard deviations between parentheses are presented

^a,b^Means with different superscripts differ significantly from each other at *p* < .05

With respect to participants’ brand memory, the analysis showed a significant effect of duration, χ^2^(2) = 7.26, *p* = .02. Logistic regression [−2 Log Likelihood = 77.09, Nagelkerke R^2^ = .14, χ^2^(3) = 7.53, *p* = .03] showed that when participants recognized the disclosure, only the 6-s disclosure resulted in greater brand recall compared to no disclosure (*b* = 1.72, *OR* = 5.59, *p* = .01), and the 3-s disclosure did not (*b* = 0.43, *OR* = 1.53, *p* = .32). Moreover, an additional logistic regression showed that the 6-s disclosure resulted in significantly higher brand recall compared to the 3-s disclosure (*b* = −1.72, *OR* = 0.18, *p* = .01). This means that when participants did recognize the disclosure, only the 6-s disclosure had a significant positive effect on brand recall.

Moreover, an ANCOVA showed no significant effects of duration on brand attitude: *F*(2, 80) = 0.12, *p* = .45, η^2^ = .00. Tests for any indirect or mediated effects of the 3- and 6-s disclosures on brand attitude via the three persuasion knowledge measures did not reveal any significant results either (see Table 3). This means that the replication among adolescents did not find any evidence for an effect of sponsorship disclosure duration on brand attitudes. Overall, this means that only H7c was supported in the subsample: disclosure duration positively affected brand memory but not persuasion knowledge or brand attitude.

## Discussion

The present study aimed to increase our understanding of the effects of sponsorship disclosure and its duration on adolescents’ (i.e., recognition of brand placement as being advertising, understanding that brand placement has a persuasive intent and critical attitude toward brand placement) and brand responses (i.e., brand memory and brand attitude). To do so, we replicated the study by Boerman et al. (2012) among adolescents aged 13–17 year old. Replicating Boerman et al. made it possible to compare the effects of sponsorship disclosure on adolescents and adults. Research among adults has shown that disclosures for brand placement can activate persuasion knowledge (i.e., people’s recognition of brand placement as being advertising, understanding of the persuasive and selling intent of brand placement and critical attitudes) and mitigate persuasion. However, based on insights on adolescent development, there are strong reasons to assume that disclosure effects on adolescents are different than for adults. These reasons have to do with adolescents’ levels of persuasion knowledge, their immature executive function skills (i.e., working memory, inhibition, and cognitive flexibility), and the importance of identity development and related susceptibility to product and brand symbolism.

### Effects on Persuasion Knowledge

Exposure to sponsorship disclosure did not affect adolescents’ recognition of the brand placement as a type of advertising or their attitudinal persuasion knowledge. However, it did increase their understanding of the persuasive intent of brand placement when they explicitly remembered seeing the disclosure. This means that only when adolescents paid thorough attention to the disclosure and processed it did it exert influence on one aspect of adolescents’ conceptual persuasion knowledge (i.e., their understanding of the persuasive intent of brand placement). Importantly, the first crucial step in mitigating effects on brand attitudes, the activation of attitudinal persuasion knowledge (Boerman et al. 2012), did not take place among adolescents.

When comparing our findings with those of Boerman et al. (2012), it is interesting to see that the level of recognition of advertising in the no-disclosure condition was already higher among adolescents (*M* = 5.50, *SD* = 1.81 in the subsample) than among adults (*M* = 5.07, *SD* = 1.66). This means that without a disclosure, adolescents in our sample seemed to be better in recognizing brand placement in a television program than adults. The disclosure did not further increase recognition of the brand placement as a type of advertising among adolescents. These effects seem to imply a ceiling effect among adolescents that was not present among adults. It seems that adolescents in general are more aware of or used to the practice of brand placement than adults.

Moreover, whereas Boerman et al. (2012) showed that sponsorship disclosure increased adults’ attitudinal persuasion knowledge (i.e., critical attitudes toward brand placement), both through conceptual persuasion knowledge and directly when the disclosure lasted for 6 s, our study showed no effects on attitudinal persuasion knowledge among adolescents. The means indicate that the levels of attitudinal persuasion knowledge are similar for adolescents (overall *M* = 3.73, *SD* = 1.18) and adults (overall *M* = 3.76, *SD* = 1.09) but that they are significantly raised by a disclosure of 6 s among adults but not among adolescents. This implies that for adolescents, a disclosure and knowing that brand placement is advertising or has a persuasive intent does not consequently lead to more critical beliefs about the advertising, whereas it does for adults.

The differences in disclosure effects between adults and adolescents may be explained by the disclosure duration. It could be that the disclosures used were not strong enough for adolescents because, due to immature executive functioning skills, they have more difficulties in focusing their attention and in processing distinct pieces of information at the same time (Best and Miller 2010; Blakemore and Choudhury 2006). The richness of information presented in a television program that also includes brand placement and disclosures may make it difficult for adolescents to process the disclosure. Disclosures that are shown longer than 6 s may be more effective in activating adolescents’ attitudinal persuasion knowledge because it gives them more opportunity to process the disclosure and think about the brand placement in a critical manner. Whereas for adults 6 s of disclosure display may be enough to activate their attitudinal persuasion knowledge, adolescents may need more time.

Together, these findings provide valuable new insights into the level of persuasion knowledge regarding brand placement among adolescents. In addition, it illuminates the role of persuasion knowledge in adolescents’ critical processing of brand placement.

### Effects on Brand Responses

The present study showed that sponsorship disclosure increased brand memory among adolescents. This result corroborates findings from previous studies including the original study by Boerman et al. (Boerman et al. 2012, 2015; Van Reijmersdal et al. 2013; Wood et al. 2008). Disclosures of sponsorship, whether remembered or not, seem to enhance the attention paid to the placed brand, increasing the level of processing and eventually brand memory.

Importantly, sponsorship disclosure did not affect brand attitudes among adolescents. There was no direct effect but also no indirect effect via conceptual or attitudinal persuasion knowledge. These findings are contrary to those found by Boerman et al. (2012). They showed that, among adults, disclosures can evoke more critical processing of brand placement, which in turn results in resistance toward persuasion as indicated by more negative attitudes toward the brand. This means that, unlike among adults, sponsorship disclosure does not mitigate persuasion among adolescents.

These findings suggest that whereas adults do alter their attitudes toward brand placement and the advertising brand when they are made aware of advertising in a program, adolescents do not. There are two reasons that may explain this. First, adolescents’ cognitive and social development can explain these differential effects of sponsorship disclosure on persuasion. Adolescents do not seem to “stop and think” critically about the brand placement due to the disclosure, probably because of their limited executive function skills (Rozendaal et al. 2011). Second, adolescents are more susceptible to consumer symbolism, materialism, and social status than adults (Moore and Stephens 1975; Solomon 1983), and this may have overruled the message in the disclosure and the activation of resistance. In other words, for adolescents, brands have an important social meaning. Many adolescents in the current study knew and watched the program *MTV Was Here*, and in general they were interested in shoes (product interest *M* = 4.15). This means that this specific program advertising a specific shoe brand may not have caused resistance but could have functioned as a signal that these shoes are considered as “cool.” Further research should compare the effects of disclosures and the activation of several elements of persuasion knowledge on persuasion for different types of programs and products.

### Effects of Disclosure Duration

Our study demonstrated that brand memory increased significantly when the disclosure was visible for 6 rather than 3 s among those adolescents who remembered seeing the disclosure. As hypothesized, the longer duration of the disclosure, which also included the brand name, offered adolescents more opportunity to process the brand. Boerman et al. (2012) showed no effect of disclosure duration on brand memory. This seems to imply that adolescents do need the extra opportunity to process the disclosure before the brand is stored in memory, whereas for adults, a 3-s disclosure is already enough.

Interestingly, disclosure duration did not affect conceptual or attitudinal persuasion knowledge or brand attitudes. With regard to attitudinal persuasion knowledge and brand attitude, there were no effects of the mere presence of disclosures, and the duration did not increase this. For these outcomes, the disclosure was just not successful, regardless of its duration. For the understanding of persuasive intent, there was a main effect of disclosure, regardless of its duration.

### Limitations and Future Research

The present study provides valuable new insights into the relative impact of sponsorships disclosure on adolescents and adults. However, more research is needed to further develop our knowledge on the effects of sponsorship disclosure on minors.

Our analyses showed that all effects held when controlled for age. Future research may include a more diverse sample of adolescents and children and examine whether there are differences in disclosure effects due to age. It could be that sponsorship disclosure has a stronger effect on older children, because their persuasion knowledge is better developed (Mangleburg and Bristol 1998; Robertson and Rossiter 1974).

In addition, the present study used only one type of disclosure. Previous research has demonstrated that the content of the disclosure determines its effects (Boerman et al. 2015; Carr and Hayes 2014; Dekker and Van Reijmersdal 2013; Tessitore and Geuens 2013). For example, stronger effects were found when not only the source but also the persuasive intent were mentioned (Dekker and Van Reijmersdal 2013). Similarly, disclosures may need to be shown for longer than 6 s to exert an effect on adolescents’ attitudinal persuasion knowledge. Future research may test whether a different content of the disclosure, a longer duration of the disclosure, or constantly showing the disclosure can help adolescents in evaluating brand placement in an adult-like manner.

Future research may also explore whether the level of attention paid to the program moderates effects of disclosures. This could, for example, be done by using an attention quiz with questions about the program content or by using visual attention measures, such as eye-tracking. Based on limited cognitive capacity theory, one may assume that disclosures are less effective for those who pay a lot of attention to the program, because these viewers have little resources left to process the disclosure (Lang 2000).

### Implications

Both theoretical and practical implications can be derived from our study. Our findings provide an empirical test of the premises of theories on children’s and adolescents’ advertising processing (Buijzen et al. 2010; Rozendaal et al. 2011). These theories, in which a developmental perspective on adult persuasion models is adopted, postulate that due to the embedded nature of advertising formats such as brand placements and immature executive functioning skills, children and adolescents are unlikely to show critical systematic levels of information processing, which our study confirms. Even with a disclosure, adolescents are not triggered to resist persuasion or to activate attitudinal persuasion knowledge.

In addition, our study seems to confirm that adolescents may be less motivated to process brand placement critically even when persuasion knowledge is activated (i.e., they recognize brand placement as a form of advertising and understand that it has a persuasive intent), probably because of the social meaning of brands, which is very informative for adolescents’ identity development (Brown 1990; John 1999; Grotevant 1987; Moore and Stephens 1975).

Practically, our study showed that a sponsorship disclosure as used in this study is hardly able to inform adolescents about the persuasive intent of brand placement. Adolescents’ understanding of the persuasive intent was only enhanced when adolescents remembered seeing the disclosure; therefore, the sponsorship disclosures were able to inform less than one-third of our sample about the *persuasive nature* of brand placement, which is dramatically low. However, our study also showed that adolescents’ recognition of advertising was already higher than that of adults when no disclosure was shown. This seems to indicate that adolescents are in less need of disclosures when recognition of advertising is concerned.

For legislators, our study implies that it may be most beneficial to use 3-s disclosures among adolescents, given the fact that this disclosure increased understanding of persuasive intent and at the same time did not result in unintended side effects (i.e., increased brand memory). However, these effects only held when adolescents remembered seeing the disclosure. To establish an adult-like critical level of processing brand placement among adolescents, the disclosures used in this study do not suffice. Rather, to help adolescents fully develop their attitudinal persuasion knowledge and enable them to use this knowledge to mitigate persuasion, educational programs or campaigns can be used *in combination with* disclosures. Such programs should be tailored to adolescents, explaining the persuasive intent of brand placement and other forms embedded advertising and its potential for biased and less trustworthy information.

For advertisers, our results imply that sponsorship disclosure does not undermine brand placement persuasion among adolescents. In fact, sponsorship disclosure even increases brand memory. In addition, understanding of persuasive intent is enhanced among those adolescents who remember seeing the disclosure, which increases the fairness of brand placement. In other words, without resulting in more negative attitudes toward brand placement or toward the placed brand, disclosures do result in more responsible advertising toward minors.

## Conclusion

This study shows that sponsorship disclosure has fundamentally different effects on adolescents than on adults. More specifically, this study shows that disclosure of brand placement has limited effects on adolescents’ persuasion knowledge: It only affects adolescents’ understanding of the persuasive intent of brand placement and not their recognition of advertising, or their critical attitudes toward brand placement. Moreover, this study shows that disclosure does not mitigate persuasion among adolescents as their attitude toward the placed brand remains unaffected. However, the disclosure did lead to higher levels of brand memory. Brand memory increased with the duration of the disclosure.

These findings add new insights to the study of adolescence and brand placement disclosures, because they imply that a unique combination of advertising characteristics and developmental characteristics determine how adolescents respond to brand placement disclosures. Adolescents are unlikely to show critical systematic levels of information processing even when a disclosure of brand placement is provided due to (a) the embedded nature of advertising formats such as brand placements, (b) adolescents’ immature executive functioning skills (Best and Miller 2010; Blakemore and Choudhury 2006), and (c) the social meaning of brands among adolescents, which is very informative for adolescents’ identity development (Brown 1990; John 1999; Grotevant 1987; Moore and Stephens 1975).

## Footnotes

1 In accordance with EU regulations (European Commission 2010), the disclosure was shown at the beginning of the program just after the introduction, in the middle of the program concurrent with the sponsored content, or just before the end of the program. Additional analyses did not reveal any significant interaction effects of the disclosure timing and duration. Therefore, we decided to leave out the factor timing in the current study.

2 Reported results are significant at *p* < .05 because *p* values are divided by two to test one-tailed.
